# Ameloblastic Fibroma of the Maxilla with Bilateral Presentation: Report of a Rare Case with Review of the Literature

**DOI:** 10.1155/2015/250713

**Published:** 2015-01-05

**Authors:** Kranti Kiran Reddy Ealla, Vijayabaskar Reddy Basavanapalli, Surekha Reddy Velidandla, Sangameshwar Manikya, Rajesh Ragulakollu, Prasanna M. Danappanavar, Vijayasree Vennila

**Affiliations:** ^1^Department of Oral and Maxillofacial Pathology, MNR Dental College and Hospital, Sangareddy, Telangana 502294, India; ^2^Department of Oral and Maxillofacial Surgery, MNR Dental College and Hospital, Sangareddy, Telangana 502294, India; ^3^Department of Pedodontics and Preventive Dentistry, KLR'S Lenora Institute of Dental Sciences, Rajahmundry, Andhra Pradesh 533294, India; ^4^Department of General Pathology, Kamineni Institute of Medical Sciences, Narketpally, Telangana 508254, India

## Abstract

Ameloblastic fibroma (AF) is an uncommon benign odontogenic tumour, with both epithelial and mesenchymal neoplastic proliferation. It occurs most frequently in the posterior region of the mandible, while its occurrence in the maxilla is extremely rare. They are usually encountered in children, emphasizing it as an important diagnostic consideration. Herein, we report the first case of a bilateral maxillary ameloblastic fibroma in a 2-year-old female child patient who presented with a chief complaint of swelling in the right mid facial region.

## 1. Introduction

Ameloblastic fibromas (AFs) are a rare variety of benign odontogenic tumors composed of proliferating odontogenic epithelium embedded in a cellular ectomesenchymal tissue resembling dental papilla [1]. It was first described by Kruse in 1891 and later classified as a separate entity by Thoma and Goldman in 1946 [2, 3]. They are frequently encountered in the posterior mandible with eighty percent cases in the second primary molar or first permanent molar region [4] and 75% associated with an impacted tooth [5]. These tumors are frequently diagnosed between the 1st and 2nd decades of life with 75% of cases being diagnosed before the age of 20 and primarily considered a tumor of childhood and adolescence. Males show a slightly higher prediction than females (M : F = 1.4 : 1) [6].

AFs usually present with a well-defined unilocular or multilocular radiolucencies [7]. Unilocular lesions are predominantly asymptomatic, while the multilocular cases are often associated with jaw swelling [8]. However, most of the cases of AFs are encountered as an incidental finding [9, 10] reiterating their radiographic significance in the differential diagnosis with entities such as dentigerous cyst, ameloblastoma, odontogenic keratocyst, and ameloblastic fibrosarcoma [11, 12].

Microscopically AFs are composed of both the epithelial and connective tissue components; the later appears to recapitulate dental papilla made up of spindled and angular cells with delicate collagen, imparting a myxomatous appearance. The epithelial component is arranged in thin branching cords or small nests with scanty cytoplasm and basophilic nuclei, while stellate reticulum like cells are common in larger nests. Mitoses are not a characteristic feature of ameloblastic fibroma [13]. In contrast to conventional ameloblastoma, the strands of AFs show double or triple layer of cuboidal cells. Numerous mitotic cells or any atypical mitotic figure if noticed suggests a malignant entity such as ameloblastic fibrosarcoma (AFS) in the differential diagnosis [14, 15].

AFs are usually treated conservatively by enucleation with curettage of the surrounding normal bone, while the aggressive lesions require a radical approach [16]. The present case reports signifies the importance of careful differential diagnosis of intrabony oral lesions with an atypical location.

## 2. Case Report

**Figure 1:**
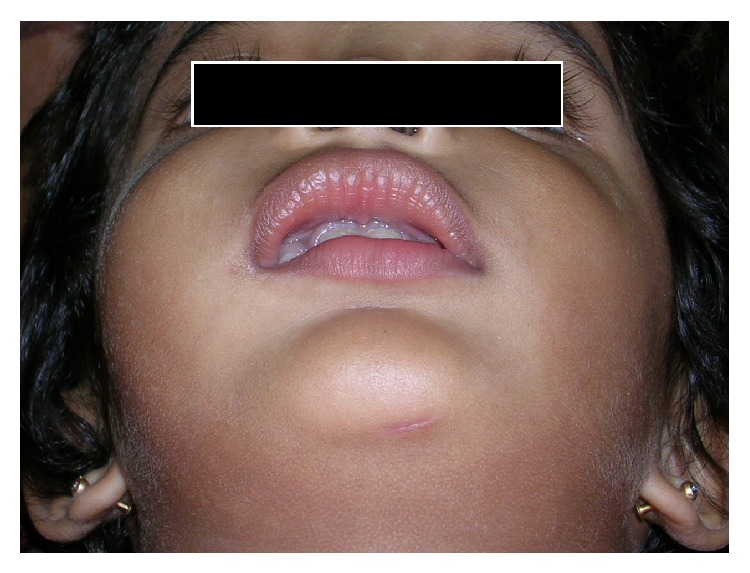
Extraoral view showing mild swelling on the right and left mid face.

A 2-year-old female child patient visited the department of oral and maxillofacial surgery with a complaint of diffuse swelling in the right mid face since one year. The swelling was progressive with gradual increase in size, consequently involving the contralateral side causing a swelling in the left mid facial region after 6 months. Both the swellings exhibited gradual increase in size with evidence of nasal blockage (Figure 1).

**Figure 2:**
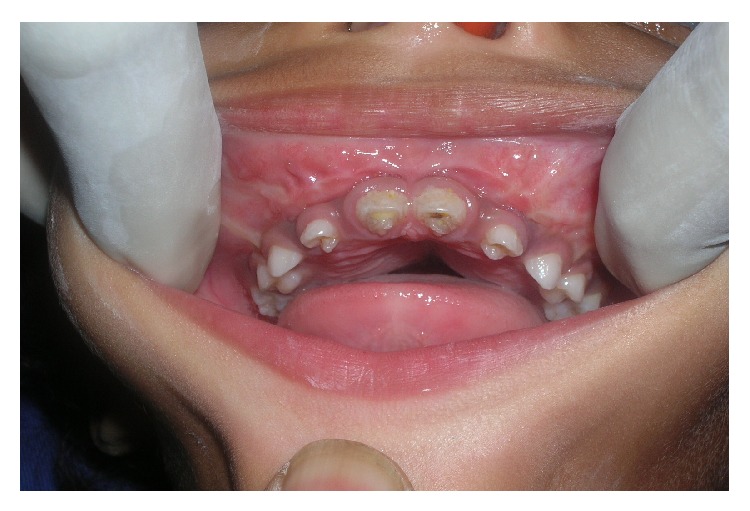
Intraoral view showing lobulated, bony hard swelling.

On examination, the swellings were diffuse extending on to the zygomatic arch, nontender with no secondary changes. Intraoral examination revealed a nontender, lobulated swelling which was firm to bony hard in consistency (Figure 2). The swelling caused labial and buccal cortical expansion bilaterally and extended up to the pterygoid plates along with palatal bone thickening. The overlying mucosa was intact. The patient's family history revealed that the elder sister, who is currently five years old, had a similar complaint 3 years ago, which was then operated, and a histopathology report of ameloblastic fibroma was rendered. Presently she is free from any recurrence.

**Figure 3:**
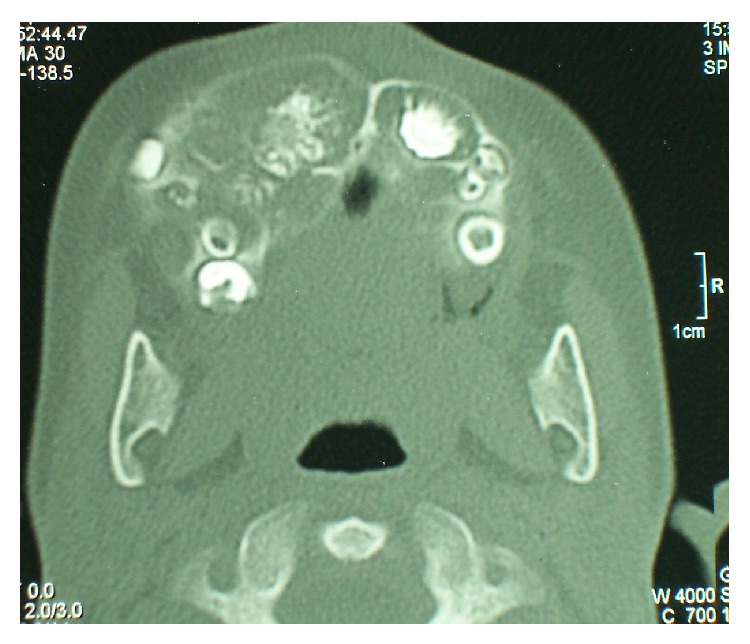
CT scan revealing hyperdense mass involving the labial cortex and pterygoid plates.

**Figure 4:**
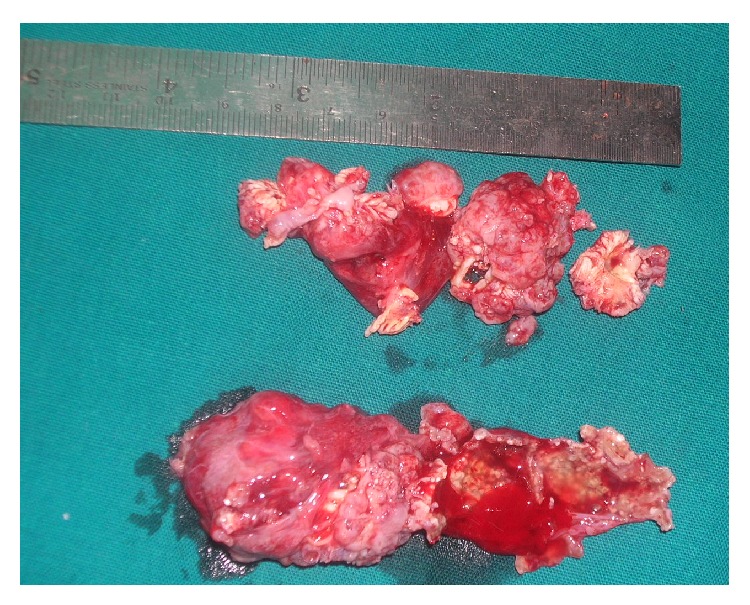
Gross picture showing a lobulated and smooth surface.

Computed tomography with 3D reconstruction was performed which revealed a hyperdense mass involving the labial and buccal cortex and extending up to the pterygoid plates. The palatal bone also showed thickening with irregular surface (Figure 3). Based on the clinical and roentgenographic findings, a presumptive preoperative diagnosis of odontogenic tumor was made. The lesion was excised and curettage of the adjacent maxillary bone was performed under general anesthesia. The surgical specimen was then sent for histopathological analysis. Macroscopically, the specimen measured 3.5 × 1.5 cm in greatest dimension and was firm in consistency with a smooth surface (Figure 4).

**Figure 5:**
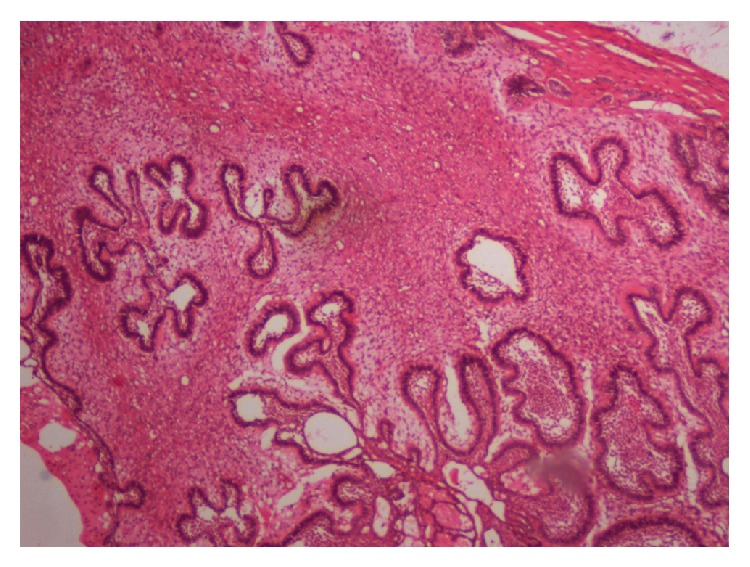
Photomicrograph showing ameloblastic islands in a cellular connective tissue stroma.

**Figure 6:**
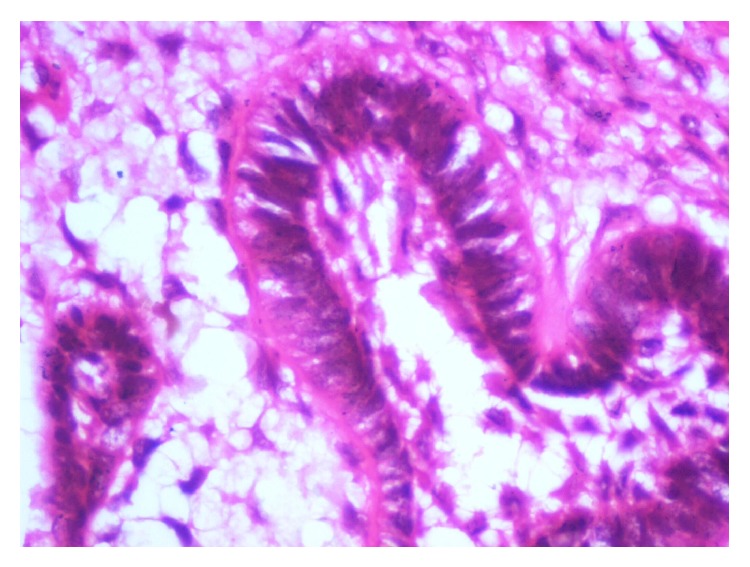
Photomicrograph showing tall columnar ameloblasts with reversal of polarity.

Microscopically, the lesion showed proliferation of strands of ameloblastic epithelial cells within a moderately cellular connective tissue stroma that closely simulates the dental papilla. The epithelial islands, nests, and strands were composed of peripheral tall columnar hyperchromatic cells exhibiting reversal of polarity and loosely arranged central cells having angular to spindle shape. The mesenchymal component comprised evenly distributed plump ovoid and stellate cells in a matrix of loose myxoid tissue (Figures 5 and 6).

## 3. Discussion

Ameloblastic fibroma is a true-mixed neoplasm of odontogenic origin with both epithelial and mesenchymal tissues [4]. These neoplasms are noticed in young patients especially in the first two decades of life [17] and mandible is considered to be the most common site of occurrence than the maxilla by a factor 3.1 [18]. Incidence of Maxillary AF is believed to be uncommon by itself; its bilateral presentation is exceedingly rare. To the best of our knowledge, this is the first case presentation of a bilateral maxillary ameloblastic fibroma.

Males are more commonly affected than females, who are usually diagnosed between the first and second decades of life frequently presenting with a painless swelling of the jaw. The present patient was only 2 years old, fitting into the normal spectrum of ameloblastic fibroma, with the youngest age reported in a seven-week-old infant [19].

However, the clinical manifestations of AF are not characteristic and the tumor is frequently observed as an incidental finding in a routine radiographic examination [20]. Normal eruption of the teeth in the affected area is usually altered with more than one-third of cases associated with an impacted tooth [21]. Radiographically they appear unilocular or multilocular with smooth well-demarcated borders [9] which are often misdiagnosed as dentigerous cyst when associated with an impacted tooth. Differential diagnosis of AF must also include entities such as ameloblastoma, odontogenic keratocyst, and ameloblastic fibrosarcoma [12, 13]. Cortical expansion of the affected bone is commonly observed [22] which was noted in the present case elucidating its true neoplastic nature.

Microscopically the epithelial component occupies the mesenchymal stroma in various patterns like thin long strands, cords, nests, or islands. Unlike the strands in ameloblastoma, the strands in AF exhibit double or triple layer of cuboidal cells [23]. The ectomesenchymal component is composed of typical plump fibroblasts with delicate collagen fibrils simulating the dental papilla [24]. The amount of cellularity differs from area to area within the same tumor and between tumors. A narrow cell-free zone bordering the epithelium and juxta-epithelial hyalinization in the connective tissue, which ultrastructurally may represent exuberant basal lamina with or without resemblance to early stage of normal odontogenesis was noted [25, 26].

AFs are classified based on histology as granular cell type, where granular cells predominate in ectomesenchyme, papilliferous with marked proliferation of the epithelium [4], ameloblastoma in association with AF and cystic ameloblastoma [21]. If dentin or enamel were noticed, they are classified as an ameloblastic fibrodentinoma or an ameloblastic fibroodontoma, respectively. Cahn and Blum proposed the continuum concept by stating that these three mixed tumours were thought to represent various stages of tooth development [27]. There stands a controversy on this spectrum of lesions whether these should be classified as different entities or represent different stages of maturation of the same entity.

The nature of AF still stands enigmatic, as there has been a long debate as to whether ameloblastic fibroma represents a hamartomous growth or is a true benign neoplasm. This controversy further attributes to the difficulties to differentiate between the histology of the neoplastic and the hamartomatous lesions with the histologic features of ameloblastic fibroma [4]. In recent times, it has been proposed that two variants of AF exist, namely, a neoplastic type with no inductive phenomenon and a hamartomatous type revealing inductive capabilities [21]. However, few authors contradicted this view and highlighted the true neoplastic nature by pointing out that AFs are seen in adults (>22 yrs) where odontogenesis is completed and tendency to recur and the recurrent cases do not show further steps of differentiation and potential to turn malignant [24].

AFs show high rate of recurrence with more than 45% turning to malignant ameloblastic fibrosarcoma [24]. In addition to detecting the mitotic figures in the histology, immunohistochemical analysis using ki-67, PCNA, and p53 labelling indices would further aid in delineating AFS from AF [28].

## 4. Conclusion

A careful treatment planning is necessary considering their recurrence rate and ability to undergo malignant transformation. As it is difficult to differentiate a hamartomatous lesion from a neoplasm based merely on histology, age of the patient should be an important consideration while deciding the treatment plan. Radical therapeutic methods should not be performed in the management of AFs in young patients [29]. Considering the age and high recurrence rate, a long-term follow-up is recommended, particularly in our case where both the siblings were diagnosed with AF necessitating future insight into the genetics.

## References

[1] Kramer I. R. H., Pindborg J. J., Shear M. (1992). The WHO histological typing of odontogenic tumours. *Cancer*.

[2] Kruse A. (1891). Ueber die Entwickelung cystischer Geschwülste im Unterkiefer. *Archiv für Pathologische Anatomie und Physiologie und für Klinische Medicin*.

[3] Thoma K. H., Goldman H. M. (1946). Odontogenic tumors—a classification based on observations of the epithelial, mesenchymal, and mixed varieties. *The American Journal of Pathology*.

[4] Reichart P. A., Philipsen H. P. (2004). Ameloblastic fibroma. *Odontogenic Tumors and Allied Lesions*.

[5] Takeda Y. (1999). Ameloblastic fibroma and related lesions: current pathologic concept. *Oral Oncology*.

[6] Cohen D. M., Bhattacharyya I. (2004). Ameloblastic fibroma, ameloblastic fibro-odontoma, and odontoma. *Oral and Maxillofacial Surgery Clinics of North America*.

[7] Slootweg P. J. (1981). An analysis of the interrelationship of the mixed odontogenic tumors—amelobastic fibroma, ameloblastic fibro-odontoma, and the odontomas. *Oral Surgery Oral Medicine and Oral Pathology*.

[8] Chen Y., Li T. J., Gao Y., Yu S. F. (2005). Ameloblastic fibroma and related lesions: a clinicopathologic study with reference to their nature and interrelationship. *Journal of Oral Pathology and Medicine*.

[9] Chen Y., Wang J.-M., Li T.-J. (2007). Ameloblastic fibroma: a review of published studies with special reference to its nature and biological behavior. *Oral Oncology*.

[10] Barnes L., Eveson J. W., Reichart P. A., Sidransky P. (2005). *Pathology and Genetics of Tumours of the Head and Neck: World Health Organization Classification of Tumours: International Histol Ogical Classification of Tumors*.

[11] Lopez R. A. G., Ortega L., Corchon M. A. G., Sandez A. B. (2003). Ameloblastic fibroma of the man dible: report of the two cases. *Medicina Oral*.

[12] Kim S. G., Jang H. S. (2002). Ameloblastic fibroma: report of a case. *Journal of Oral and Maxillofacial Surgery*.

[13] Kobayashi K., Murakami R., Fujii T., Hirano A. (2005). Malignant transformation of ameloblastic fibroma to ameloblastic fibrosarcoma: case report and review of the literature. *Journal of Cranio-Maxillofacial Surgery*.

[14] Tomich C. E. (1999). Benign mixed odontogenic tumors. *Seminars in Diagnostic Pathology*.

[15] Regezi J. A., Sciubba J. J., Jordan R. C. (2003). *Oral Pathology Clinical Pathologic Correlations*.

[16] Kulkarni R. S., Sarkar A., Goyal S. (2013). Recurrent ameloblastic fibroma: report of a rare case. *Case Reports in Dentistry*.

[17] Shafer W. G., Hine M. K., Levy B. M., Rajendran R., Sivapathasundharam B. (2006). Cysts and tumors of odontogenic origin. *Shafers Textbook of Oral Pathology*.

[18] Jindal C., Bhola R. S. (2011). Ameloblastic fibroma in six-year-old male: hamartoma or a true neoplasm. *Journal of Oral and Maxillofacial Pathology*.

[19] Mosby E. L., Russell D., Noren S., Barker B. F. (1998). Ameloblastic fibroma in a 7-week-old infant: a case report and review of the literature. *Journal of Oral and Maxillofacial Surgery*.

[20] da Costa D. O., Alves A. T., Calasans-Maia M. D., da Cruz R. L., Lourenço S. D. Q. C. (2011). Maxillary ameloblastic fibroma: a case report. *Brazilian Dental Journal*.

[21] Mcguinness N. J., Faughnan T., Bennani F., Connolly C. E. (2001). Ameloblastic fibroma of the anterior maxilla presenting as a complication of tooth eruption: a case report. *Journal of Orthodontics*.

[22] Vasconcelos B. C. E., Andrade E. S. S., Rocha N. S., Morais H. H. A., Carvalho R. W. F. (2009). Treatment of large ameloblastic fibroma: a case report. *Journal of Oral Science*.

[23] Reichart P. A., Philipsen H. P. (2004). *Ameloblastic Fibroma. Odontogenic Tumors and Allied Lesions*.

[24] Neville B. W., Damm D. D., Allen C. M., Bouquot J. E. (2011). *Oral and Maxillofacial Pathology*.

[25] Farman A. G., Gould A. R., Merrell E. (1986). Epithelium—connective tissue junction in follicular ameloblastoma and ameloblastic fibroma: an ultrastructural analysis. *International Journal of Oral and Maxillofacial Surgery*.

[26] van Wyk C. W., van der Vyver P. C. (1983). Ameloblastic fibroma with dentinoid formation/immature dentinoma. *Journal of Oral Pathology & Medicine*.

[27] Cahn L. R., Blum T. (1952). Ameloblastic odontoma: case report critically analyzed. *Journal of Oral Surgery*.

[28] Bernardes V. F., Gomes C. C., Gomez R. S. (2012). Molecular investigation of ameloblastic fibroma: how far have we gone?. *Head and Neck Oncology*.

[29] Nelson B. L., Folk G. S. (2009). Ameloblastic fibroma. *Head and Neck Pathology*.

